# Slit Binding via the Ig1 Domain Is Essential for Midline Repulsion by *Drosophila* Robo1 but Dispensable for Receptor Expression, Localization, and Regulation *in Vivo*

**DOI:** 10.1534/g3.115.022327

**Published:** 2015-09-10

**Authors:** Haley E. Brown, Marie C. Reichert, Timothy A. Evans

**Affiliations:** Department of Biological Sciences, University of Arkansas, Fayetteville, Arkansas 72701

**Keywords:** axon guidance, midline repulsion, roundabout, slit, *Drosophila*

## Abstract

The midline repellant ligand Slit and its Roundabout (Robo) family receptors constitute the major midline repulsive pathway in bilaterians. Slit proteins produced at the midline of the central nervous system (CNS) signal through Robo receptors expressed on axons to prevent them from crossing the midline, and thus regulate connectivity between the two sides of the nervous system. Biochemical structure and interaction studies support a model in which Slit binding to the first immunoglobulin-like (Ig1) domain of Robo receptors activates a repulsive signaling pathway in axonal growth cones. Here, we examine the *in vivo* functional importance of the Ig1 domain of the *Drosophila* Robo1 receptor, which controls midline crossing of axons in response to Slit during development of the embryonic CNS. We show that deleting Ig1 from Robo1 disrupts Slit binding in cultured *Drosophila* cells, and that a Robo1 variant lacking Ig1 (Robo1^∆Ig1^) is unable to promote ectopic midline repulsion in gain-of-function studies in the *Drosophila* embryonic CNS. We show that the Ig1 domain is not required for proper expression, axonal localization, or Commissureless (Comm)-dependent regulation of Robo1 *in vivo*, and we use a genetic rescue assay to show that Robo1^∆Ig1^ is unable to substitute for full-length Robo1 to properly regulate midline crossing of axons. These results establish a direct link between *in vitro* biochemical studies of Slit–Robo interactions and *in vivo* genetic studies of Slit-Robo signaling during midline axon guidance, and distinguish Slit-dependent from Slit-independent aspects of Robo1 expression, regulation, and activity during embryonic development.

## Introduction

### Slits and Robos in midline axon guidance

In animals with bilateral symmetry, coordination between the two sides of the body depends on the proper establishment of neuronal connectivity across the midline of the central nervous system (CNS). During embryonic development, neuronal axons must correctly choose whether to remain on the same side of the body (ipsilateral) or to cross the CNS midline and establish connections with contralateral targets (Evans and Bashaw 2010a). The repellant ligand Slit and its Roundabout (Robo) family receptors regulate axon guidance in the CNS: Slit proteins are produced by cells at the midline and signal through Robo receptors expressed on the surface of axonal growth cones to prevent midline crossing of axons. Slit and Robo constitute the major midline repellant pathway in bilaterians, and disruption of Slit-Robo signaling causes ectopic midline crossing phenotypes in the CNS of a broad range of animals, including vertebrates, insects, nematodes, and planarians (Kidd *et al.* 1998a; Zallen *et al.* 1998; Fricke *et al.* 2001; Long *et al.* 2004; Cebrià *et al.* 2007; Evans and Bashaw 2012).

### Slit–Robo interaction studies

Slit and Robo were first identified as a ligand–receptor pair in *Drosophila*, and the expression patterns of Slit and Robo orthologs in vertebrates immediately suggested an evolutionarily conserved role in regulating midline crossing of axons (Kidd *et al.* 1998a; Brose *et al.* 1999; Kidd *et al.* 1999). In *trans*-species binding experiments in cultured cells, *Drosophila* Slit could bind to mammalian Robo receptors (rat Robo1 and Robo2), and human Slit2 could also bind to *Drosophila* Robo1 (Brose *et al.* 1999). These results suggested a deep conservation of not only the functional roles of Slit and Robo but also the molecular mechanism of Slit–Robo interaction. Consistent with this, a number of structure–function studies revealed that the biochemical interaction between Slits and Robos from vertebrates and flies alike depends on the leucine-rich repeat (LRR) region of Slit, most importantly the LRR2 (D2) domain, and the extracellular immunoglobulin-like (Ig) domains of Robo receptors, specifically Ig1 and Ig2 (Chen *et al.* 2001; Battye *et al.* 2001; Nguyen Ba-Charvet *et al.* 2001; Liu *et al.* 2004; Howitt *et al.* 2004). Crystal structure and site-directed mutagenesis studies of the *Drosophila* Robo1/Slit and human Robo1/Slit2 complexes demonstrated that the molecular interaction between Slit D2 and Robo Ig1 is highly conserved and suggested that the Ig1 domain of Robo receptors is the primary Slit-binding domain in both insects and vertebrates (Morlot *et al.* 2007; Fukuhara *et al.* 2008). However, the *in vivo* functional importance of Ig1 has not yet been investigated in any system.

### Midline crossing in *Drosophila*

In *Drosophila*, the single Slit ortholog signals through two Robo receptor paralogs (Robo1 and Robo2) to regulate midline crossing of axons in the embryonic CNS. Robo1 (also known simply as Robo) is the primary Slit receptor in *Drosophila*, whereas Robo2 plays a more minor role in midline repulsion. In *robo1* null mutants, strong ectopic crossing is observed in every segment of the embryonic CNS, while in *robo2* mutant embryos mild ectopic crossing is observed in a minority of segments. Simultaneous removal of *robo1* and *robo2* reproduces the severe midline collapse phenotype observed in *slit* mutants, where the majority of CNS axons enter the midline and never leave due to a complete absence of midline repulsion (Rajagopalan *et al.* 2000; Simpson *et al.* 2000).

Although Robo1 protein is constitutively expressed in nearly all embryonic neurons, the majority of axons in the fly embryonic ventral nerve cord will cross the midline once and remain on the contralateral side (Kidd *et al.* 1998a; Rickert *et al.* 2011). As commissural axons approach and cross the midline, premature sensitivity to Slit is prevented by the endosomal sorting receptor Commissureless (Comm), which limits the amount of Robo1 that reaches the growth cone surface (Kidd *et al.* 1998b; Keleman *et al.* 2002, 2005) and Robo2, which acts nonautonomously to antagonize Slit–Robo repulsion to promote midline crossing (Evans *et al.* 2015). After midline crossing, *comm* transcription is extinguished and Robo1 levels increase on the growth cone surface, restoring Slit sensitivity and preventing recrossing. Regulation of Robo1 trafficking by Comm has been proposed to account for the observation that antibody staining against Robo1 strongly labels longitudinal axon pathways but is nearly undetectable on commissures in wild-type embryos (Kidd *et al.* 1998a), although there is some evidence to suggest that exclusion of Robo1 from commissural segments may be independent of Comm sorting (Gilestro 2008).

Although the genetic relationship between *slit* and *robo1* has been well characterized *in vivo*, and the biochemical nature of Slit–Robo interaction has been studied intensively *in vitro*, a disconnect remains between these *in vitro* interaction studies and *in vivo* functional studies. Current models predict that a Robo1 receptor that cannot bind Slit should not be able to repel axons *in vivo*, but this prediction has not been directly tested. Further, it remains unknown whether Slit binding is important for expression or regulation of Robo1 *in vivo*. Here, we bridge this gap by reporting the expression, regulation, and activity of a Slit binding-deficient form of Robo1 in the *Drosophila* embryonic CNS. Using gain-of-function and genetic rescue approaches, we show that deleting the Slit-binding Ig1 domain of Robo1 does not affect its expression, axonal localization, or Comm-dependent regulation *in vivo*, and we demonstrate for the first time that Slit binding via Ig1 is absolutely required for Robo1’s midline repulsive role in an endogenous expression context in intact animals.

## Materials and Methods

### Molecular biology

#### pUAST cloning:

Robo coding sequences were cloned into a pUAST vector (p10UASTattB) including 10xUAS and an attB site for ΦC31-directed site-specific integration. All p10UASTattB constructs include identical heterologous 5′ UTR and signal sequences (derived from the *Drosophila* *wingless* gene) and an N-terminal 3xHA tag. Robo1^∆Ig1^ includes amino acids 153–1395 of Robo1 (relative to Genbank reference sequence AAF46887).

#### robo1 rescue construct cloning:

The *robo1* genomic rescue construct is based on the work of Spitzweck *et al.* (2010). Upstream and downstream flanking sequences from the *robo1* gene were amplified by PCR and cloned into a plasmid containing attB and *mini-white* sequences. An in-frame 4xHA tag followed by a *Bam*HI restriction site was inserted between the upstream flanking region (2385 bp beginning with GAATTCCTCCAGGAAACTGT and ending with TCCTACTCCTTTCAGGCCAG) and downstream flanking region (2192 bp beginning with TGTTTGAGACTCTCCGAATA and ending with CTTGGCAGTAACGGTCTCCG). Robo coding sequences were amplified via PCR with *Bgl*II sites added to both primers, digested with *Bgl*II, and cloned into the *Bam*HI-digested backbone. Robo1 proteins produced from this construct include the endogenous Robo1 signal peptide, and the 4xHA tag is inserted directly upstream of the first Ig domain (Ig1 in Robo1; Ig2 in Robo1^∆Ig1^).

### Genetics

The following *Drosophila* mutant alleles were used: robo1^1^ (also known as robo^GA285^) and eg^MZ360^ (eg-GAL4). The following *Drosophila* transgenes were used: P{GAL4-elav.L}3 (elavGAL4), P{UAS-TauMycGFP}III, P{10UAS-HARobo1}86Fb, P{10UAS-HARobo1^∆Ig1^}86Fb, P{UAS-CommHA}, P{robo1::HArobo1}, and P{robo1::HArobo1^∆Ig1^}. Transgenic flies were generated by BestGene Inc (Chino Hills, CA) using ΦC31-directed site-specific integration into attP landing sites at cytological position 86F8 (for UAS-Robo constructs) or 28E7 (for robo1 genomic rescue constructs). All crosses were carried out at 25°.

### Slit binding assay

S2R+ cells were cultured at 25° in Schneider’s media plus 10% fetal calf serum. To assay Slit binding, cells were plated on poly-L-lysine–coated coverslips in six-well plates (Robo-expressing cells) or untreated six-well plates (Slit-expressing cells) at a density of 1–2×10^6^ cells/ml and transfected with pRmHA3-GAL4 (Klueg *et al.* 2002) and HA-tagged pUAST-Robo or untagged pUAST-Slit plasmids using Effectene transfection reagent (Qiagen). GAL4 expression was induced with 0.5 mM CuSO_4_ for 24 hr, and then Slit-conditioned media was harvested by adding heparin (2.5 ug/ml) to Slit-transfected cells, which were incubated at room temperature for 20 min with gentle agitation. Robo-transfected cells were incubated with Slit-conditioned media at room temperature for 20 min and then washed with PBS and fixed for 20 min at 4° in 4% formaldehyde. Cells were permeabilized with PBS plus 0.1% Triton X-100 and then stained with antibodies diluted in PBS plus 2 mg/ml BSA. Antibodies used were: mouse anti-SlitC (DSHB #c555.6D, 1:50); rabbit anti-HA (Covance #PRB-101C-500, 1:2000); Cy3-conjugated goat anti-mouse (Jackson #115-165-003, 1:500), and Alexa 488-conjugated goat anti-rabbit (Jackson #111-545-003, 1:500). After antibody staining, coverslips with cells attached were mounted in Aquamount. Confocal stacks were collected using a Leica SP5 confocal microscope and processed by Fiji/ImageJ (Schindelin *et al.* 2012) and Adobe Photoshop software.

### Immunohistochemistry

*Drosophila* embryo collection, fixation, and antibody staining were performed as previously described (Patel 1994). The following antibodies were used: FITC-conjugated goat anti-HRP (Jackson Immunoresearch #123-095-021, 1:100); mouse anti-Fasciclin II [Developmental Studies Hybridoma Bank (DSHB) #1D4, 1:100]; mouse anti-βgal (DSHB #40-1a, 1:150); mouse anti-Robo1 (DSHB #13C9, 1:100); rabbit anti-GFP (Invitrogen #A11122, 1:1000); mouse anti-HA (Covance #MMS-101P-500, 1:1000); Cy3-conjugated goat anti-mouse (Jackson #115-165-003, 1:1000); Alexa 488–conjugated goat anti-rabbit (Jackson #111-545-003, 1:500); and HRP-conjugated goat anti-mouse (Jackson #115-035-003, 1:250). Embryos stained with HRP-conjugated antibodies were developed by incubation with Stable Diaminobenzidine (DAB) solution (Invitrogen) according to the manufacturer’s instructions. Embryos were genotyped using balancer chromosomes carrying *lacZ* markers or by the presence of epitope-tagged transgenes. Nerve cords from embryos of the desired genotype and developmental stage were dissected and mounted in 70% glycerol/PBS. Fluorescent confocal stacks were collected using a Leica SP5 confocal microscope and processed by Fiji/ImageJ (Schindelin *et al.* 2012) and Adobe Photoshop software. DIC images were acquired using a Zeiss Axioskop 2 microscope attached to a Canon EOS Rebel T2i digital camera and processed by Adobe Photoshop software.

### Data availability

*Drosophila* strains are available upon request.

## Results

### Deletion of the Ig1 domain prevents Slit–Robo1 interaction in cultured *Drosophila* cells

Biochemical interaction studies *in vitro* have established a model of Slit–Robo repulsive signaling in which Slit binding to Robo receptors via the Robo Ig1 domain is a key event in the repulsive signaling pathway that repels ipsilateral and postcrossing commissural axons from the CNS midline. However, Slit–Robo interaction studies to date have been carried out with purified protein fragments *in vitro* and have not addressed the importance of Robo Ig1 for Slit binding in a cellular context or the predicted functional requirement for the Robo Ig1 domain *in vivo*.

**Figure 1:**
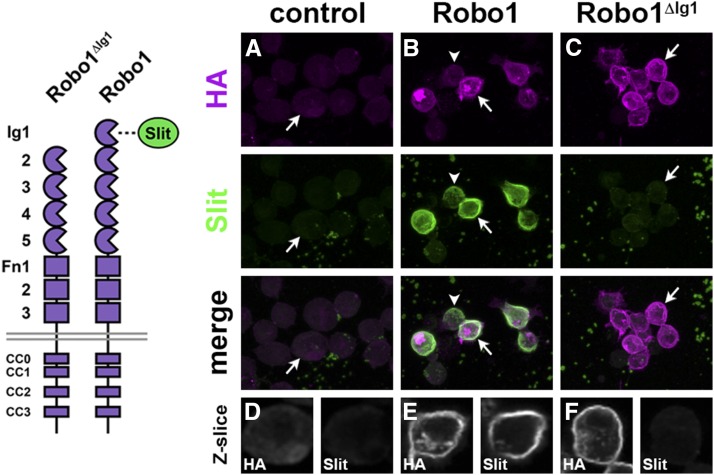
Deletion of the Robo1 Ig1 domain prevents Slit–Robo1 interaction in cultured *Drosophila* cells. *Drosophila* S2R+ cells were transfected with the indicated HA-tagged *UAS-Robo* transgenes, incubated with Slit-conditioned media, and then stained with anti-HA (magenta) and anti-Slit (green) antibodies. Slit does not bind to mock-transfected cells that do not express transgenic Robo1 (A), but binds robustly to cells expressing a full-length Robo1 transgene (B). The level of Slit binding correlates with the level of Robo1 expression, as cells expressing lower levels of Robo1 also exhibit weaker Slit binding (arrowhead). Cells expressing transgenic Robo1^ΔIg1^ exhibit similar levels of Slit binding to control cells (C). (A–C) Confocal max projections through the entire cells; (D–F) single confocal Z-slices through the cells indicated with arrows in (A–C). Robo1^ΔIg1^ is properly localized at the plasma membrane, similar to Robo1 (compare HA in E and F), indicating that deletion of Ig1 does not disrupt expression of Robo1 at the cell surface. Schematics of the tested Robo receptor variants are shown at left.

To evaluate the importance of Slit binding for the *in vivo* activity of Robo1, we first examined whether deletion of the Robo1 Ig1 domain would abolish Slit binding in a cellular context using transmembrane receptors expressed at the surface of *Drosophila* cells. To this end, we incubated cultured *Drosophila* S2R+ cells expressing HA-tagged transgenic Robo1 or Robo1^ΔIg1^ with conditioned media harvested from cells expressing full-length Slit. We found that Slit bound robustly to cultured *Drosophila* cells expressing transgenic full-length Robo1, but interacted only weakly with untransfected cells or cells expressing Robo1^ΔIg1^ (Figure 1). Importantly, Robo1^ΔIg1^ protein was expressed at levels similar to full-length Robo1 and was properly localized to the plasma membrane, as assayed by anti-HA staining of transfected cells. Thus, deletion of the Ig1 domain from Robo1 strongly abrogates Slit binding but does not affect expression or membrane localization of the receptor in cultured cells.

### Transgenic Robo1^ΔIg1^ is localized to axons *in vivo* and does not prevent midline crossing when misexpressed

**Figure 2:**
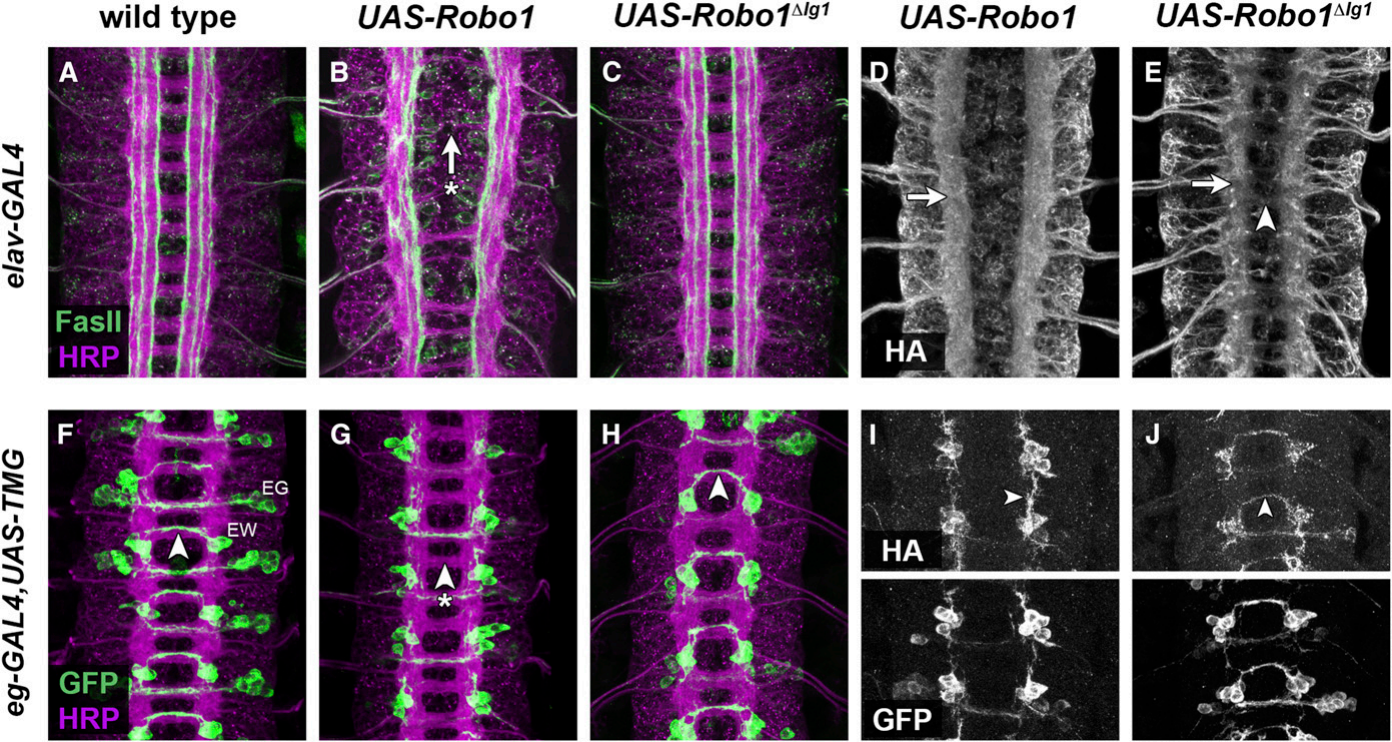
Transgenic Robo1^ΔIg1^ is localized to axons *in vivo*, is downregulated on commissures, and does not prevent midline crossing when misexpressed. (Top) Stage 16 *Drosophila* embryos carrying *elav-GAL4* and the indicated HA-tagged *UAS-Robo* transgenes stained with anti-HRP (magenta; labels all axons) and anti-FasII (green; labels a subset of longitudinal axon pathways) (A–C) or anti-HA (D, E). (A) The axon scaffold forms normally in embryos carrying *elav-GAL4* alone. (B) Misexpression of Robo1 with *elav-GAL4* inhibits midline crossing, leading to thin or absent commissures (arrow with asterisk). FasII-positive longitudinal pathways also appear disorganized. (C) Misexpression of Robo1^ΔIg1^ with *elav-GAL4* does not affect midline crossing or FasII pathway formation, and the nerve cord looks wild-type. (D, E) Transgenic Robo1 and Robo1^ΔIg1^ are both expressed on longitudinal axons (arrows). Robo1^ΔIg1^ levels are much lower on commissures (arrowhead in E). (Bottom) Stage 15 *Drosophila* embryos carrying *eg-GAL4*, *UAS-TauMycGFP* (*TMG*), and the indicated HA-tagged *UAS-Robo* transgenes stained with anti-HRP (magenta) and anti-GFP (green) (F–H) or anti-HA and anti-GFP (I, J). (F) *eg-GAL4* labels the EG and EW neurons, whose axons cross the midline in the anterior and posterior commissures, respectively. In wild-type embryos, the EW axons cross the midline in every segment (arrowhead). (G) Misexpression of Robo1 with *eg-GAL4* prevents the EW axons from crossing the midline (arrowhead with asterisk). (H) EW axons are not prevented from crossing the midline by misexpression of Robo1^ΔIg1^ (arrowhead). Anti-HA staining in (I) and (J) shows that both transgenes are expressed on EW axons (arrowheads); GFP staining of the same segments is shown below for comparison. For quantification of EW crossing defects in the genotypes shown in (F–H), see Table 1.

To compare the expression, localization, and activity of Robo1 and Robo1^ΔIg1^ *in vivo*, we generated transgenic *Drosophila* lines with our HA-tagged *UAS-Robo1* and *UAS-Robo1^ΔIg1^* constructs. To ensure equivalent expression levels, both transgenes were inserted in the same genomic location using ΦC31-directed site-specific integration (insertion site 86Fb). We used the GAL4/UAS system to express these receptors in the *Drosophila* embryo, either broadly in all embryonic neurons (using *elav-GAL4*) or in a restricted subset of commissural neurons (EG and EW neurons using *eg-GAL4*) (Figure 2). We used antibodies against horseradish peroxidase (HRP, which recognizes a pan-neural epitope in *Drosophila* and labels all of the axons in the embryonic CNS) (Snow *et al.* 1987; Haase *et al.* 2001) and Fasciclin II (FasII, which labels a subset of longitudinal axon pathways) (Grenningloh *et al.* 1991) to examine the embryonic ventral nerve cord under conditions of pan-neural Robo1 misexpression with *elav-GAL4* (Figure 2, A–C). We used an anti-GFP antibody to label the cell bodies and axons of the EG and EW commissural neurons in embryos carrying *eg-GAL4* and *UAS-TauMycGFP (UAS-TMG)* (Figure 2, F–H).

As expected, pan-neural misexpression of Robo1 strongly inhibited midline crossing in the embryonic ventral nerve cord, producing thin or absent commissures in nearly 100% of segments in *elav-GAL4/UAS-Robo1* embryos (Figure 2B). We also observed a strong disorganization of FasII-positive longitudinal axon pathways in these embryos, which may be a secondary consequence of disrupted midline crossing, or may reflect interference with the lateral positioning activities of Robo2 and Robo3 due to the high levels of Robo1 misexpression. Antibody staining against the N-terminal HA tag confirmed that transgenic Robo1 was localized to axons in these embryos (Figure 2D). Consistent with our observations in cultured cells (see above), deletion of the Ig1 domain did not affect the expression levels or localization of Robo1 in embryonic neurons, but completely disrupted its ability to promote ectopic midline repulsion (Figure 2, C and E). Embryos misexpressing Robo1^ΔIg1^ with elav-GAL4 were indistinguishable from wild-type embryos (compare Figures 2, A and C), suggesting that preventing Slit binding by deleting Ig1 removes Robo1’s midline repulsive activity. Consistent with this, we found that misexpression of Robo1 with *eg-GAL4* prevents midline crossing of the commissural EW axons in 97% of segments, whereas equivalent expression of Robo1^ΔIg1^ had no effect on EW midline crossing (Figure 2, F–H; Table 1).

**Table 1:** EW axon midline crossing defects caused by Robo1 misexpression

Genotype	% Segments with EW Noncrossing	N (Segments/Embryos)
*eg-GAL4,UAS-TMG/+*	0.0	104/13
*eg-GAL4,UAS-TMG/UAS-Robo1*	97.1	104/13
*eg-GAL4,UAS-TMG/UAS- Robo1^ΔIg1^*	2.9	103/13

Stage 15 and 16 embryos carrying *eg-GAL4*, *UAS-TauMycGFP (UAS-TMG)*, and the indicated *UAS-Robo1* transgenes were stained with anti-GFP. Abdominal segments from dissected ventral nerve cords were scored for midline crossing of the GFP-positive EW axons.

### Transgenic Robo1^ΔIg1^ is unable to rescue midline crossing in *robo1* mutants

**Figure 3:**
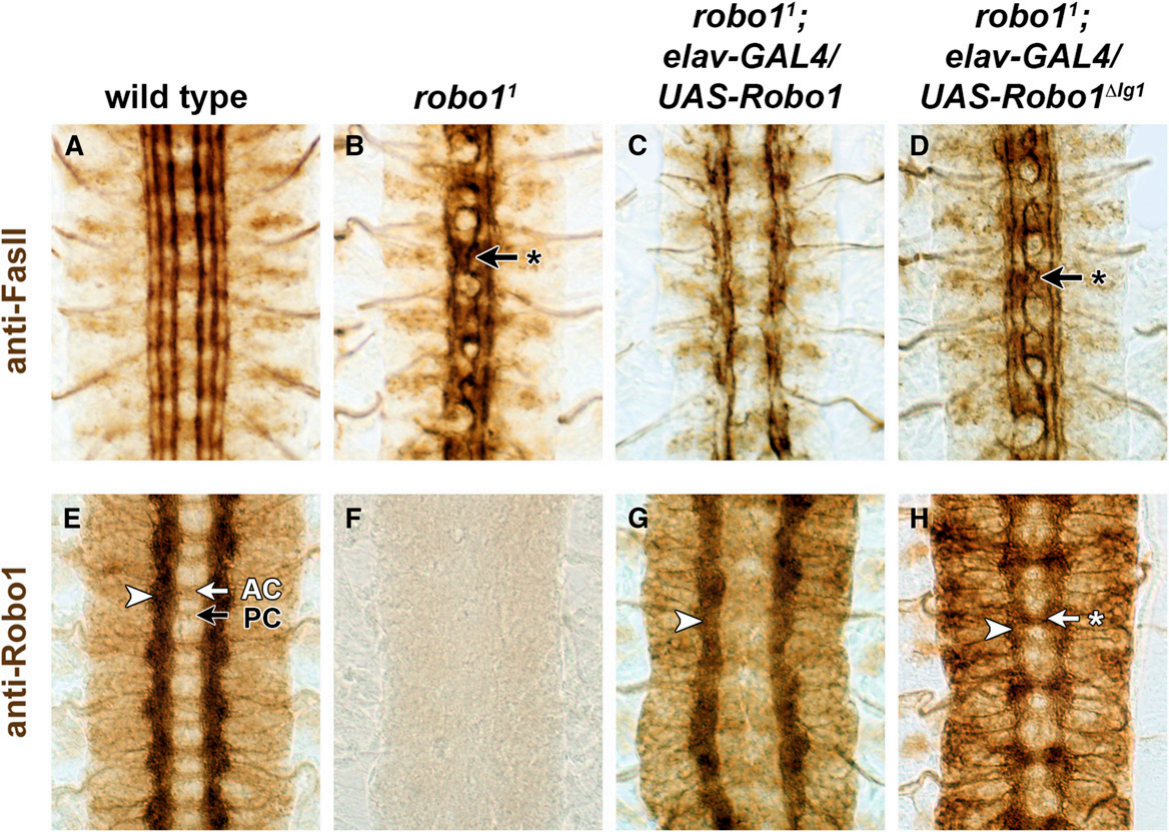
Pan-neuronal expression of Robo1^ΔIg1^ is unable to rescue midline crossing in *robo1* mutants. Stage 16 *Drosophila* embryos stained with anti-FasII (top) or anti-Robo1 (bottom). In wild-type embryos, FasII-positive axons do not cross the midline (A), and Robo1 protein is localized to longitudinal axon pathways (arrowhead) but excluded from commissural axon segments in both the anterior commissure (AC, white arrow) and posterior commissure (PC, black arrow) (E). (B) In homozygous *robo1* null mutants, FasII-positive axons ectopically cross the midline in 100% of segments (arrow with asterisk). Robo1 protein is undetectable in these embryos (F). When Robo1 expression is restored in neurons in *robo1* mutants carrying *elavGAL4* and *UAS-Robo1*, FasII axons no longer cross the midline (C), and Robo1 protein is again detectable on longitudinal pathways (G). Commissure formation is strongly inhibited in these embryos, and FasII pathways are disorganized. (D) Neuronal expression of Robo1^ΔIg1^ does not rescue midline repulsion in *robo1* null mutants. (H) Robo1^ΔIg1^ protein is expressed on longitudinal pathways in *robo1* mutant embryos carrying *elavGAL4* and *UAS-Robo1^ΔIg1^* (arrowhead), and is also detectable on axons as they cross the midline, especially in the anterior commissure (arrow with asterisk). For quantification of ectopic crossing phenotypes in the genotypes shown in (A–D), see Table 2.

The gain-of-function experiments described above suggest that Robo1^ΔIg1^ is unable to signal midline repulsion in *Drosophila* neurons when ectopically expressed. To test whether the Robo1 Ig1 domain is required for Robo1’s normal role in midline repulsion, we performed a rescue assay using our *UAS-Robo1* and *UAS-Robo1^ΔIg1^* transgenes in *robo1* null mutants. As *robo1* is normally broadly expressed in embryonic neurons, we used *elav-GAL4* to drive *UAS-Robo1* expression in all embryonic neurons in *robo1* null mutant embryos and assayed midline repulsion using anti-FasII antibody (Figure 3, A–D). We also used anti-Robo1 antibody to assay expression of endogenous Robo1 and transgenic Robo1 and Robo1^ΔIg1^ in our wild-type, *robo1* mutant, and genetic rescue backgrounds (Figure 3, E–H). Transgenic Robo1 and Robo1^ΔIg1^ proteins both include the epitope recognized by the 13C9 anti-Robo1 antibody.

FasII-positive axons do not cross the midline in late-stage wild-type *Drosophila* embryos (Figure 3A), and endogenous Robo1 protein is detectable on longitudinal axons in these embryos (Figure 3E). In *robo1* null mutants, Robo1 protein expression is undetectable (Figure 3F), and FasII-positive axons cross the midline in every segment (Figure 3B; Table 2). When we restored transgenic Robo1 expression in neurons of *robo1* mutants carrying *elav-GAL4* and *UAS-Robo1*, FasII-positive axons no longer crossed the midline (Figure 3C; Table 2) and Robo1 protein expression was again detectable on noncrossing axons (Figure 3G). Forcing high-level expression of Robo1 in all neurons in *robo1* mutants caused additional guidance defects, including disruption of normal commissure formation and disorganization of longitudinal axon pathways (compare Figure 3A and Figure 3C), as observed with Robo1 misexpression in wild-type embryos (Figure 2B). In contrast, pan-neural expression of Robo1^ΔIg1^ did not restore midline repulsion in a *robo1* mutant background, and ectopic crossing of FasII-positive axons in *robo1^1^/robo1^1^*; *elav-GAL4/UAS-Robo1^ΔIg1^* embryos looked identical to *robo1^1^/robo1^1^* null mutants (Figure 3D). Importantly, the inability of Robo1^ΔIg1^ to rescue midline crossing is not due to mislocalization of the receptor, as Robo1^ΔIg1^ expression was readily detectable on axons in *robo1^1^/robo1^1^*; *elav-GAL4/UAS-Robo1^ΔIg1^* embryos (Figure 3H).

**Table 2:** Ectopic midline crossing defects in *robo1* mutant and rescue backgrounds

Genotype	% Segments with Ectopic FasII Crossing	N (Segments/Embryos)
*robo1^1^/+*	5.2	96/12
*robo1^1^/robo1^1^*	100	80/10
***GAL4/UAS rescue***		
*robo1^1^/robo1^1^*; *elav-GAL4/UAS-Robo1*	3.8	104/13
*robo1^1^/robo1^1^*; *elav-GAL4/UAS-Robo1^ΔIg1^*	100	104/13
***robo1 genomic rescue***		
*robo1^1^*, *robo1*::*robo1/robo1^1^*, *robo1*::*robo1*	2.9	104/13
*robo1^1^*, *robo1*::*robo1^ΔIg1^/*	100	104/13
*robo1^1^*, *robo1*::*robo1 ^ΔIg1^*		

Stage 16 and 17 embryos were stained with anti-FasII and abdominal segments from dissected ventral nerve cords were scored for ectopic midline crossing of FasII-positive axons.

### The Robo1 Ig1 domain is not required for normal expression and localization

**Figure 4:**
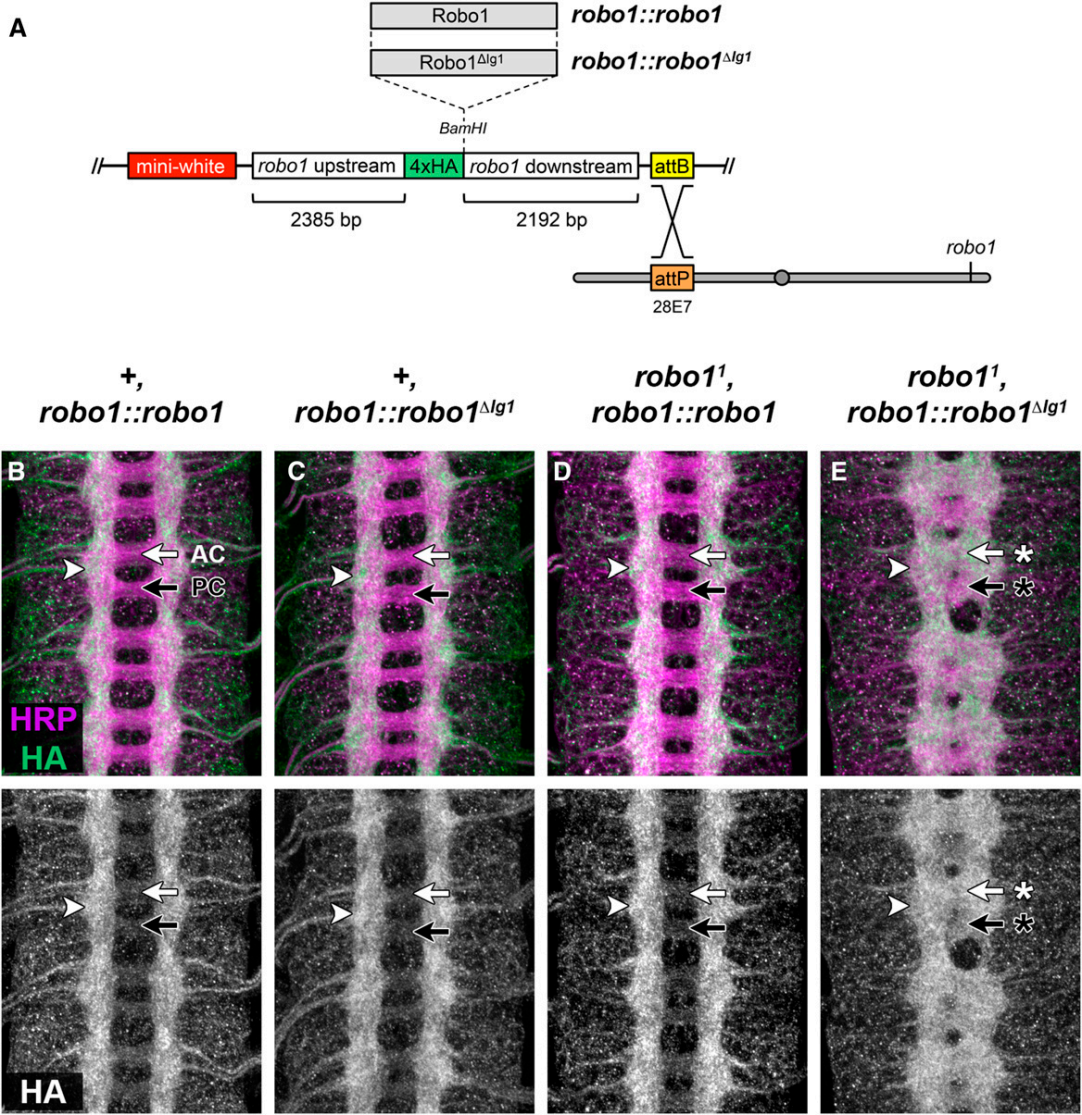
Expression of Robo1 and Robo1^ΔIg1^ proteins via a *robo1* genomic rescue transgene. (A) Schematic of *robo1* rescue construct. Open reading frames are cloned into the *BamHI* restriction site in-frame with the N-terminal 4xHA epitope tag and are expressed under the control of *robo1* genomic regulatory sequences. Rescue constructs carrying full-length Robo1 or Robo1^ΔIg1^ coding sequences were inserted into the same genomic landing site at cytological position 28E7. *robo1* mutations were introduced onto these chromosomes via meiotic recombination. (B–E) Stage 16 embryos stained with anti-HRP (magenta) and anti-HA (green) antibodies. Bottom images show HA channel alone from the same embryos. (B, C) In a wild-type background, HA-tagged full-length Robo1 (B) or Robo1^ΔIg1^ (C) proteins expressed from the *robo1* rescue transgene are localized to longitudinal axon pathways (arrowhead) and are excluded from commissural axon segments in both the anterior commissure (AC, white arrow) and posterior commissure (PC, black arrow). The HA staining pattern in both embryos closely matches the expression of endogenous Robo1 protein in wild-type embryos (compare to Figure 3E). (D) Embryos homozygous for a null allele of *robo1* and carrying two copies of the *robo1*::*robo1* rescue construct display a wild-type axon scaffold, and the distribution of HA-tagged Robo1 is the same as in a wild-type background. (E) Homozygous *robo1* mutants carrying two copies of the *robo1*::*robo1^ΔIg1^* transgene exhibit a *robo1* loss of function phenotype, with thickened commissures and longitudinal pathways that form closer to the midline. HA-tagged Robo1^ΔIg1^ is detectable on longitudinal pathways (arrowhead) and also on both commissures (arrows with asterisks), although Robo1^ΔIg1^ levels appear higher on AC (white arrow with asterisk) than PC (black arrow with asterisk).

The above experiments comparing the expression and activity of Robo1 and Robo1^ΔIg1^ rely on GAL4/UAS-based misexpression, which uncouples *robo1* expression from the factors that normally control its expression pattern and levels. As seen above, this can lead to confounding effects such as inhibition of normal commissure formation and FasII pathway disorganization in our GAL4-based rescue experiments. To compare our receptor variants under conditions that more closely match *robo1*’s endogenous expression pattern and levels, we generated a *robo1* genomic rescue construct that uses regulatory sequences derived from the endogenous *robo1* locus to control expression of HA-tagged Robo1 or Robo1^ΔIg1^ cDNAs (Figure 4A). Both rescue constructs (*robo1*::*robo1* and *robo1*::*robo1^ΔIg1^*) contain identical upstream and downstream regulatory sequences, and we inserted both transgenes into the same genomic location to ensure equivalent expression levels (insertion site 28E7). A similar construct was previously used to examine the ability of chimeric Robo1/Robo3 receptors to rescue *robo1*-dependent midline repulsion (Spitzweck *et al.* 2010).

We found that the HA-tagged Robo1 protein expressed from our *robo1* rescue construct *(robo1*::*robo1)* closely reproduced the normal Robo1 expression pattern in the embryonic CNS: it was detectable across the entire width of the longitudinal connectives and was strongly downregulated on commissural axon segments (Figure 4B). Notably, expression of the HA-Robo1 transgene in a wild-type background (which already carries two functional copies of the endogenous *robo1* gene) did not produce any discernible gain-of-function effects, even when it was also present in two copies (*i.e.*, in *+*, *robo1*::*robo1* homozygous embryos). We observed an identical expression pattern with the HA-Robo1^ΔIg1^ transgene *(robo1*::*robo1^ΔIg1^)* in a wild-type background, indicating that deleting the Ig1 domain does not interfere with the expression, localization, or regulation of Robo1 when expressed in its endogenous pattern in an otherwise wild-type nervous system. Expression of Robo1^ΔIg1^ did not induce any apparent dominant negative effects, as the axon scaffold appeared normal in *+*, *robo1*:: *robo1^ΔIg1^* homozygous embryos when visualized with anti-HRP antibody staining (Figure 4C).

### Regulation of Robo1^ΔIg1^ by Comm

**Figure 5:**
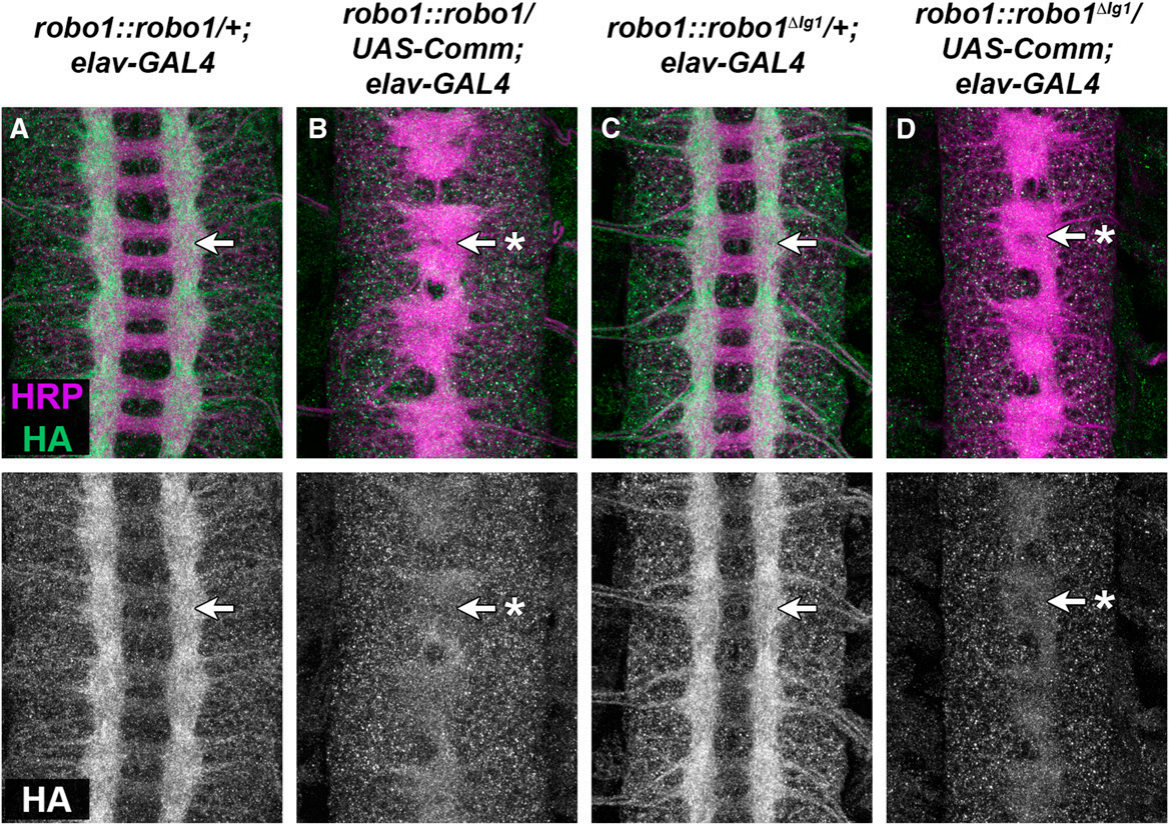
Robo1^ΔIg1^ is sensitive to downregulation by Comm. (A–D) Stage 16 embryos stained with anti-HRP (magenta) and anti-HA (green) antibodies. Bottom images show HA channel alone from the same embryos. Embryos carrying one copy of the *robo1*::*robo1* transgene (A) display normal expression of the HA-tagged Robo1 receptor (arrow), whereas sibling embryos carrying one copy of *robo1*::*robo1* along with *UAS-Comm* (B) display a strong reduction in HA staining along with a corresponding increase in midline crossing, leading to a *slit*-like midline collapse phenotype (arrow with asterisk). Levels of Robo1^ΔIg1^ expressed from the *robo1*::*robo1^ΔIg1^* transgene are similarly reduced in the presence of ectopic Comm expression (D) compared to sibling embryos without *UAS-Comm* (C). Pairs of sibling embryos shown here (A–B and C–D) were stained in the same tube and imaged under identical conditions to allow an accurate comparison of HA levels between embryos. All embryos (A–D) carry two copies of *elav-GAL4*.

Deleting the Ig1 domain from Robo1 does not appear to affect its expression or localization on axons and its clearance from commissures in otherwise wild-type embryos (see Figure 4C), suggesting that this receptor is properly regulated by Comm. This is consistent with previous studies that identified the peri-membrane region of Robo1 as the region responsible for Comm-dependent sorting of Robo1 (Gilestro 2008). To directly test if Robo1^ΔIg1^ is susceptible to regulation by Comm *in vivo*, we misexpressed Comm using *elav-GAL4* in embryos carrying *robo1*::*robo1* or *robo1*::*robo1^ΔIg1^* transgenes and examined expression of the transgenic receptor proteins using anti-HA. Because Comm is normally expressed only transiently in commissural neurons as their axons are crossing the midline, forcing high-level expression of Comm in all neurons leads to a strong reduction in Robo expression and a corresponding increase in midline crossing, phenocopying *robo1* or *slit* mutants depending on the level of ectopic Comm expression (Kidd *et al.* 1998b; Keleman *et al.* 2002; Gilestro 2008). We observed a strong reduction in neuronal HA staining in embryos carrying either rescue construct along with *elav-GAL4* and *UAS-Comm* compared to embryos carrying the rescue constructs with *elav-GAL4* alone. In addition, we observed thickened commissures consistent with an increase in midline crossing due to downregulation of endogenous Robo1 in these embryos (Figure 5). These results demonstrate that deleting the Ig1 domain of Robo1 does not disrupt Comm’s ability to regulate the receptor in embryonic neurons.

### Robo1^ΔIg1^ cannot rescue midline repulsion in *robo1* mutants

Next, we introduced a null mutation in the endogenous *robo1* locus (*robo1^1^*) onto the chromosomes carrying the Robo1 or Robo1^ΔIg1^ transgenes to examine their ability to rescue midline repulsion in a *robo1* null background. Homozygous *robo1* null embryos carrying two copies of the Robo1 rescue transgene *(robo1^1^*, *robo1*:: *robo1)* exhibited a wild-type axon scaffold, and expression of the Robo1 transgene was the same as in a wild-type background (Figure 4D). In contrast, Robo1^ΔIg1^ was unable to rescue midline repulsion in the absence of endogenous *robo1*, and *robo1^1^*, *robo1*:: *robo1^ΔIg1^* homozygous embryos phenocopied *robo1* null mutants (Figure 4E). In this background, anti-HA staining detected Robo1^ΔIg1^ protein on commissural axon segments, especially in the anterior commissure.

**Figure 6:**
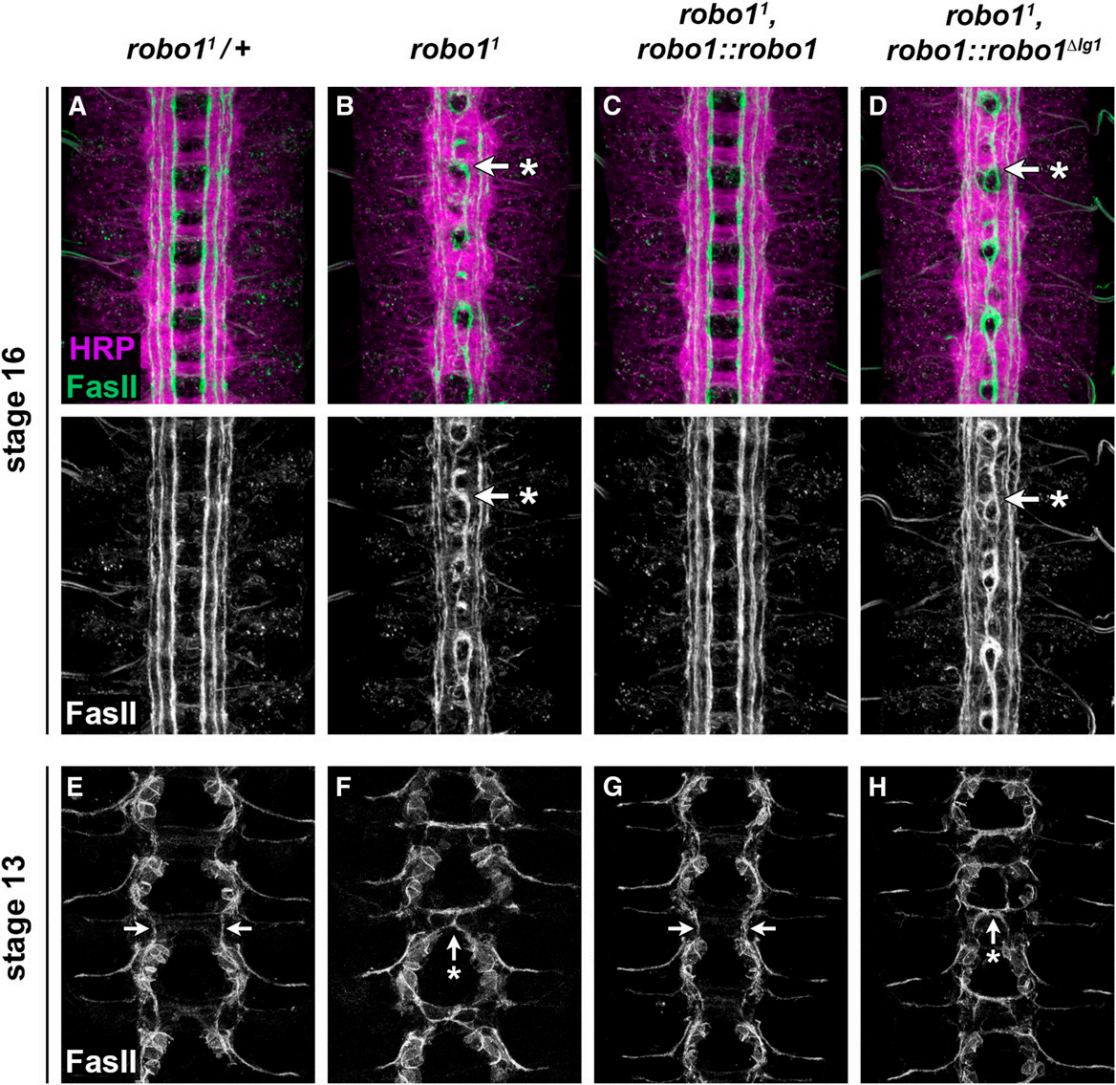
Robo1^ΔIg1^ cannot rescue midline crossing defects in *robo1* mutants. (A–D) Stage 16 embryos stained with anti-HRP (magenta) and anti-FasII (green) antibodies. Lower images show FasII channel alone from the same embryos. FasII-positive axons cross the midline inappropriately in every segment in *robo1* null mutants (B, arrow with asterisk). This phenotype is completely rescued by a *robo1* genomic rescue transgene expressing full-length Robo1 protein (C) but is not rescued by an equivalent rescue transgene expressing Robo1^ΔIg1^ (D). (E–H) Stage 13 embryos stained with anti-FasII to examine the trajectory of the pCC axon, which pioneers the medial FasII pathway. In heterozygous *robo1/+* embryos (E) or *robo1* null mutants rescued by Robo1 (G), the pCC axon extends anteriorly and does not cross the midline (arrows in E and G). In *robo1* null mutants, the pCC axon inappropriately crosses the midline and fasciculates with its contralateral homolog (F, arrow with asterisk). Ectopic crossing of pCC is not rescued by expression of Robo1^ΔIg1^ (H, arrow with asterisk). For quantification of ectopic crossing phenotypes in the genotypes shown in (A–D), see Table 2.

To more closely assess midline repulsion in our rescue backgrounds, we examined FasII-positive axon pathways, which provide a more sensitive readout of midline repulsion and can reveal more subtle ectopic crossing events that may be undetectable when examining the entire axon scaffold with anti-HRP. FasII-positive axons do not cross the midline in wild-type embryos, but a subset of these axons cross the midline ectopically in every segment in *robo1* mutants (Figure 6, A and B). We found that the Robo1 rescue transgene was able to restore wild-type levels of midline repulsion to FasII-positive axons in *robo1* null mutant embryos (Figure 6C). In contrast, the Robo1^ΔIg1^ transgene had no effect on the ectopic midline crossing caused by the *robo1* mutation, and FasII crossing defects in *robo1^1^*, *robo1*:: *robo1^ΔIg1^* embryos were indistinguishable from *robo1^1^* homozygous embryos (Figure 6D; Table 2).

The pCC neuron pioneers the medial FasII axon pathway (the pCC pathway), and its axon is labeled by the anti-FasII antibody at stage 13. In wild-type embryos, the pCC axon remains ipsilateral, but in *robo1* mutants it inappropriately extends across the midline to fasciculate with its contralateral homolog (Seeger *et al.* 1993). Consistent with our rescue results in late-stage embryos, we found that the ipsilateral projection of pCC was restored in stage 13 *robo1* mutants by our Robo1 rescue transgene, but expression of Robo1^ΔIg1^ did not prevent pCC from crossing the midline (Figure 6, E–H).

Thus, at the levels of gross morphology of the axon scaffold, a subset of longitudinal pathways, and a single ipsilateral axon, we observe that replacing the endogenous Robo1 protein with a variant that is unable to bind Slit completely eliminates its ability to regulate midline crossing of axons in the *Drosophila* embryonic CNS. Importantly, deleting the Ig1 domain from Robo1 did not detectably alter its expression or localization in embryonic neurons, confirming the specificity of this alteration and demonstrating that the expression and localization of Robo1 *in vivo* is independent of its ability to interact with Slit.

## Discussion

We have examined the functional importance of the Ig1 domain of the *Drosophila* Robo1 axon guidance receptor for Slit binding, *in vivo* expression and regulation, and repulsive axon guidance in the *Drosophila* embryonic CNS. In the context of an otherwise full-length receptor expressed at the membrane of cultured *Drosophila* cells, deleting the Ig1 domain from Robo1 strongly reduced Slit binding. Using GAL4/UAS-based misexpression, we found that Robo1 Ig1 is not required for receptor expression or axonal localization *in vivo*, but it is essential for midline repulsive signaling. Finally, using a genomic rescue construct to reproduce the endogenous expression pattern of *robo1*, we found that a version of Robo1 lacking the Ig1 domain (Robo1^ΔIg1^) is properly expressed in embryonic neurons, is excluded from commissural axon segments like full-length Robo1, and is subject to Comm-dependent downregulation, but cannot signal midline repulsion in response to Slit. Our results establish a direct connection between *in vitro* structural and protein–protein interaction studies of Slit and Robo and *in vivo* genetic studies of Slit–Robo signaling and represent the first *in vivo* investigation of the functional importance of Slit binding via Robo1 Ig1.

### Ig1 is required for Slit binding by Robo1 expressed at the membrane of *Drosophila* cells

Previous Slit–Robo binding studies used purified protein fragments or cell extracts to map the Robo-interacting region of Slit and the Slit-interacting region of Robo. The consensus from these studies is that the main interaction between Slits and Robos is mediated by the Ig1 domain of Robo receptors and the LRR2 (D2) domain of Slits (Chen *et al.* 2001; Battye *et al.* 2001; Nguyen Ba-Charvet *et al.* 2001; Howitt *et al.* 2004; Morlot *et al.* 2007; Fukuhara *et al.* 2008; Evans *et al.* 2015), although at least one study suggests that the Ig2 domain of human Robo1 may also contribute to Slit binding (Liu *et al.* 2004). Here, we show that in an otherwise full-length Robo1 receptor expressed at the surface of cultured *Drosophila* cells, deleting the Ig1 domain from Robo1 strongly disrupts Slit binding. These results confirm the importance of Ig1 for Slit binding in a cellular context and demonstrate that, in the absence of Ig1, none of the remaining sequences within Robo1’s extracellular region (including the four other structurally related Robo1 Ig domains) can confer a detectable level of Slit binding.

### Slit binding by Robo1 Ig1 is required for midline repulsion but not receptor expression or regulation *in vivo*

Using GAL4/UAS-based gain-of-function assays, GAL4/UAS-based rescue assays, and genomic fragment rescue assays, we show that removing the Ig1 domain from Robo1 (and thus preventing it from binding Slit) completely disrupts its ability to signal midline repulsion and prevent axons from crossing the midline of the embryonic CNS. In otherwise wild-type embryos, GAL4/UAS-based misexpression of Robo1 either broadly in all neurons or in subsets of commissural neurons strongly inhibits midline crossing of axons. Presumably, the high levels of Robo1 expression produced by the GAL4/UAS system overwhelm the normal negative regulatory factors (for example, Comm and Robo2) that prevent Robo1 from signaling during midline crossing of commissural axons. We note, however, that under conditions of pan-neural misexpression with *elav-GAL4*, Robo1^ΔIg1^ protein expression is still strongly downregulated on the crossing portions of commissural axons (see Figure 2E), suggesting that at least a portion of the misexpressed protein is subject to normal regulation.

We used anti-HA antibodies to confirm expression of Robo1 and Robo1^ΔIg1^ transgenic protein on EW axons in combination with *eg-GAL4*. Notably, we found that Robo1^ΔIg1^ was detectable on the midline crossing portions of EW axons. We hypothesize that the high levels of expression driven by GAL4/UAS may overwhelm the normal (Comm-dependent or Comm-independent) downregulation of Robo1 and allow a portion of the transgenic protein to reach the surface of crossing EW axons. The fact that misexpression of Robo1 prevents EW crossing would also presumably require saturation of normal regulatory mechanisms. We were not able to examine Robo1 expression on crossing EW axons (because they do not cross the midline under conditions of Robo1 misexpression), but we could detect expression of transgenic Robo1 on EW axons as they extended ipsilaterally toward the next anterior segment (Figure 2, I and J).

Notably, we did not observe any gain of function effects in embryos carrying two copies of our *robo1*::*robo1* genomic rescue transgene along with two wild-type copies of the endogenous *robo1* locus. In these embryos, Robo1 expression levels should be twice as high as in wild-type embryos, but apparently this is not enough to overwhelm normal regulation by Comm and/or Robo2. We also did not observe any dominant-negative effects of our *robo1*::*robo1^ΔIg1^* transgene, even when it was present in two copies, suggesting that the presence of Robo1^ΔIg1^ proteins at the surface of ipsilateral and postcrossing commissural axons does not interfere with the normal midline repulsive activity of endogenous Robo1 receptors.

HA-tagged Robo1^ΔIg1^ protein expressed from our *robo1*:: *robo1^ΔIg1^* transgene was restricted to longitudinal axon segments in a wild-type background, but when the endogenous *robo1* gene was disrupted we detected strong expression of Robo1^ΔIg1^ on axons as they crossed the midline (see Figure 4E). Similarly, transgenic Robo1^ΔIg1^ protein expressed from our *UAS-Robo1^ΔIg1^* transgene was excluded from commissures when expressed in otherwise wild-type embryos but was detectable on commissures when expressed in *robo1* mutant embryos (see Figure 3H). We interpret this as retention of Robo1^ΔIg1^ on ectopically crossing ipsilateral or recrossing commissural axons that are misguided as a result of loss of *robo1* (but do not express Comm), but we cannot formally rule out the alternative interpretation that this reflects mislocalization of Robo1^ΔIg1^ to normally crossing commissural axons in the absence of endogenous Robo1.

### *In vivo* roles of Robo1 domains other than Ig1

Although a number of downstream factors and regulatory components that participate in or influence Slit–Robo1 repulsion have been identified in *Drosophila* (Bashaw *et al.* 2000; Fan *et al.* 2003; Lundström *et al.* 2004; Hu *et al.* 2005; Yang and Bashaw 2006; Garbe *et al.* 2007; Coleman *et al.* 2010), the precise mechanism by which Robo1 transmits the Slit signal across the membrane, and what structural or stoichiometric changes might occur in Robo1 in response to Slit binding are not well understood (Hohenester 2008). Here, we have shown that the Ig1 domain is absolutely required for repulsive signaling by Robo1 *in vivo*. In addition to Ig1, seven other distinct domains are present in the extracellular region of Robo1 (Ig2-5 and Fn1-3); which, if any, of these are required for Slit binding, *in vivo* regulation of Robo1, or midline repulsive signaling? A comprehensive structure–function study examining the functional importance of each individual domain will increase our understanding of how the different regions of Robo1 contribute to its *in vivo* role in midline repulsion and its other developmental roles. *Drosophila* Robo2, a paralog of Robo1, regulates a diverse array of axon guidance outcomes in fly embryos and each role appears to involve a distinct combination of extracellular and cytoplasmic sequences within Robo2, including Ig1, Ig2, and Ig3 (Evans and Bashaw 2010b; Santiago *et al.* 2014; Evans *et al.* 2015). It will be interesting to learn whether such domain-dependent multi-functionality is unique to Robo2 or might be shared among *Drosophila* Robo receptors.

### Slit dependence of other developmental roles of Robo receptors

In addition to their role in midline repulsion of axons in the embryonic CNS, *Drosophila* Robo receptors also regulate a number of other developmental outcomes. Robo1 regulates embryonic muscle migration (Kramer *et al.* 2001), migration of embryonic chordotonal sensory neurons (Kraut and Zinn 2004), guidance and targeting of dendrites in the embryo and adult (Godenschwege *et al.* 2002; Furrer *et al.* 2007; Dimitrova *et al.* 2008; Mauss *et al.* 2009), and midline crossing of gustatory receptor neuron axons in the adult fly (Mellert *et al.* 2010). In each of these contexts, Robo1 has been assumed (and in some cases demonstrated) to act in response to Slit; the reagents generated here will allow a comprehensive dissection of which of Robo1’s developmental roles are Slit-dependent in future studies. We have recently demonstrated that Robo2’s noncell-autonomous role in promoting midline crossing is at least partially independent of its ability to bind Slit via its Ig1 domain (Evans *et al.* 2015). Ongoing studies in our laboratory are using similar approaches to distinguish between Slit-dependent and Slit-independent mechanisms for other axon guidance roles of *Drosophila* Robo receptors, for example, Robo2’s and Robo3’s roles in mediolateral positioning of longitudinal axon pathways.
